# Presentation of enthesitis-related arthritis and juvenile-onset spondyloarthritis: a cross-sectional study in a pediatric and adult clinic

**DOI:** 10.1186/s42358-024-00378-8

**Published:** 2024-05-08

**Authors:** Sean Hideo Shirata Lanças, Matheus Zanata Brufatto Furlan, Taciana de Albuquerque Pedrosa Fernandes, Sula Glaucia Lage Drumond, Claudia Saad Magalhães

**Affiliations:** 1 Rheumatology Division, Botucatu Medical School, São Paulo State University (UNESP), Sao Paulo, Brazil; 2 Pediatric Rheumatology Division, Botucatu Medical School, São Paulo State University (UNESP), Sao Paulo, Brazil

**Keywords:** Juvenile idiopathic arthritis, Enthesitis-related arthritis, Spondyloarthritis, Spondyloarthropathy, Classification, Outcome

## Abstract

**Background:**

Juvenile idiopathic arthritis (JIA) comprises a whole spectrum of chronic arthritis starting before 16 years of age. The study aims to explore the clinical and demographic descriptors, treatment, and disease progression of enthesitis-related arthritis (ERA) in comparison with juvenile-onset spondyloarthritis (SpA).

**Methods:**

Cross-sectional analysis of consecutive patients in two dedicated clinics, with a single visit and retrospective case-notes review. Arthritis, enthesitis and sacroiliitis were evaluated by scoring disease activity and damage. Continuous variables were reported by median, interquartile range; categorical variables were reported by the frequency comparison of the two groups.

**Results:**

Thirty-three cases were included, being 23 (69.7%) with ERA. The median age at diagnosis was 12.5 y (SpA) vs. 9 y (ERA) (*p* < 0.01); the time from symptom onset to diagnosis was 5.5 y (SpA) vs. 1.5 y (ERA) (*p* < 0.03). In both groups, the predominant presentation was a single joint or < 5 lower limb joints and asymmetric involvement, with a high frequency of enthesitis. There was a higher frequency of mid-tarsal and ankle synovitis in the ERA group and hip involvement in those with SpA. The comparison of the frequency of spine symptoms at presentation, 30% SpA vs. 21.7% ERA (*p* = 0.7), was not significant, and radiographic progression to spinal involvement occurred in 43.5% of ERA patients. The median time for spinal progression and age at onset was 2.2 and 12 y for ERA, and 4 and 16.5 y for SpA, respectively. Activity and damage scores were not significantly different between the groups. Treatment comparison resulted in 91.3% of ERA and 100% SpA being treated, predominantly with NSAIDs in both groups, followed by DMARDs and biologics, with a higher frequency of biologics in SpA.

**Conclusion:**

The main differences were the late diagnoses of SpA, and the hip and spine involvement, with higher frequency of biologic treatment in juvenile-onset SpA compared to ERA.

## Background

Juvenile idiopathic arthritis (JIA) comprises the spectrum of chronic arthritis, with a duration of more than 6 weeks, starting before the 16th birthday, with variable expression of symptoms, joint involvement and outcome, in particular with disease progression and persistence of activity and damage accrual in adult life [1]. There are many different JIA classifications based on clinical descriptors and joint count. More recently, with the knowledge of genetics and pathogenesis [2, 3], other classification system proposals and nomenclature reviews are in progress [4–6]. The current classification adopted worldwide is the *International League of Associations for Rheumatology* classification criteria (ILAR-1995), revised in 2001 [7], where mutually exclusive categories, by clinical and laboratory characteristics, are the main grouping factors. However, overlap in these categories was described, and the continuity of care in adult clinics might harmonize different perspectives of age-related expression of common pathogenesis subgroups. In the current classification, childhood exclusive presentations include early onset oligoarticular forms and those with a *continuum* spectrum of adult presentation of spondyloarthropathies [6]. A new classification proposal project by the *Pediatric Rheumatology Trials Organization* (PRINTO) will collect a worldwide scale inception cohort [8].

The enthesitis-related arthritis (ERA) subgroup characterizes an association with HLA-B27, enthesitis, asymmetric lower limb arthritis, and sacroiliac and spine involvement, represented in variable frequency in different populations, with an average of 5–10% of JIA patients, with the genetic background remarked by family history of Spondyoarthritis or related disorders associated with positive HLA-B27, male gender predominance (7:1) and onset after 6 years of age [9–11]. These characteristics are very similar to those of the spondyloarthritis (SpA) group. Enthesitis is one of the hallmarks of clinical features, presenting as inflammation of the attachment site of tendons, ligaments, joint capsules and fascia to the bone, that is seen in about 50% of cases. Other common features are the spine and hips involvement, as well as shoulder and sacroiliac joint involvement in 1/3, 1/10 and 1/3 of the patients, respectively. Such characteristics and particular joint involvement make ERA distinct from all other JIA subtypes.

The ERA classification criteria have been revised over time with some debate around the current ILAR classification and previous classifications of juvenile spondyloarthropathies (SpA), and the main criticism is in fact that it does not cover the whole spectrum of SpA [12–14]. Given the gaps of the previous and current classification, the PRINTO group proposed renaming it to “enthesitis-related arthritis/spondyloarthritis” [8], expanding the concept of spine involvement, as well as the concept of early presentation with no radiographic changes. In such a way, defining more homogeneous groups of patients followed in continuity from the pediatric to adult clinics would also favor specific treatment development [15, 16].

There is no consensus about the continuous spectrum or different age-related expressions of the same disease with common pathogenic and genetic features. ERA comprises a less frequent subtype of JIA, possibly less recognized in early young age, remaining a diagnosis and classification challenge for children and adolescents, where up to 20–40% of spine involvement cases might present with no symptoms, being recognized only by magnetic resonance imaging (MRI) [10, 11].

To better define ERA and juvenile-onset SpA similarities and differences, we aimed at exploring the clinical and demographic features as well as outcomes in a small series on follow-up in two dedicated clinics of pediatric and adult rheumatology.

## Methods

A cross-sectional assessment of pediatric and adult patients, diagnosed and followed under standard care, in a tertiary Rheumatology referral center. An independent assessment was conducted by the investigator, in addition to a retrospective case-notes review. Signed informed consent or proxy-signed consent and assent forms were obtained. It was approved by the institutional ethics committee under the number 4.520.156 on February, 2021.

The inclusion criteria were: current age from 6 to 40 years; previous diagnosis of ERA by the assistant physician and established by ILAR classification criteria [7]; or previous diagnosis of SpA presenting radiographic axial disease, or not, by the ASAS criteria [17], with onset of symptoms before 18y, regardless current age; both with at least 6 months of follow-up time in one of two dedicated clinics. The pediatric and adult clinics are based in the same hospital on different days of the week. Pediatric cases not fulfilling ERA by ILAR criteria, adult cases not fulfilling ASAS spondyloarthritis criteria, and less than 6 months of follow-up were exclusion criteria.

Standardized data collection included the descriptors of the PRINTO criteria [8], clinical, demographic and chronological descriptors, arthritis, enthesitis, spinal and sacroiliac involvement, laboratory tests, and previous and current treatment. All evaluations for arthritis joint count, active enthesitis sites and clinical signs of sacroiliitis were conducted by the same physician. Subjective symptoms and overall pain and wellbeing were scored by 0–10 visual analog scale (VAS) for spine pain, arthritis and nocturnal spinal pain. Disease activity was scored by the *Juvenile Arthritis Disease Activity Score* (JADAS10) [18], *Juvenile Spondyloarthritis Disease Activity Index* (JSpADA) [19], *Maastricht Ankylosing Spondylitis Enthesitis Score* (MASES), *Leeds Enthesitis Index* (LEI), *and Spondyloarthritis Research Consortium of Canada* (SPARCC) [20]. For structural damage assessment, the *Juvenile Arthritis Damage Index* (JADI), articular (A) and extra-articular (E) forms that were developed for JIA [21] were used. For spine radiographic damage signs, the New York criteria for plain radiographs was used, as recommended by the *Assessment in Spondyloarthritis International Society* (ASAS) [22]; MRI sacroiliac joints were analyzed according to *Outcomes Measures in Rheumatology* (OMERACT) recommendations [23]. Patients were enrolled consecutively as a convenience sample.

Summary data for comparison were grouped according to primary diagnosis of ERA and juvenile-onset SpA. Descriptive statistics of continuous variables by median and interquartile interval (IQ) and frequency and percentage for categorical variables were carried out. Joint involvement was categorized as peripheral joints and spine/SI joints; lower and upper limbs; and symmetry. The number of swollen and painful joints were considered continuous variable. The cumulative distribution of arthritis sites was considered from the disease onset to the last assessment, during the cross-sectional study point. Comparisons between the two groups were performed with the Mann‒Whitney statistical test for continuous variables and Fisher’s exact test for categorical variables. A p value < 0.05 was considered significant.

## Results

Between January 2021 and March 2022, 36 consecutive cases fulfilling the inclusion criteria were identified. Of those, one refused to participate, and 2 had less than 6 months of follow-up; therefore, 33 participants were included, with 23 (69.7%) from the pediatric clinic and 10 (30.3%) from the SpA adult clinic, with 54.5% male and 81.8% white. In Table 1, we present their sociodemographic descriptions, and in Tables 2 and 3, we present clinical characteristics at disease onset and follow-up for both groups, respectively.

**Table 1:** Comparison of demographic profile, frequency of positive HLA-B27 and uveitis in ERA and Juvenile-onset SpA groups of patients

Variables	ERA (*n* = 23)	SpA (*n* = 10)	*p* value
Age, median (IQR)	13 (11-14.5)	26.5 [19–36]	0.01
Male, n (%)	12 (52.2)	6 (60)	0.7
White, n (%)	18 (78.2)	9 (90)	0.5
Family history of SpA, n (%)	6 [26]	4 [40]	0.7
Positive HLA B-27 ‡. n (%)	5 (31.2)	3 (33.3)	0.62
Uveitis, n (%)	3 [13]	2 [20]	0.6
Comorbidities, n (%)	10 (43.5)	5 (50)	1.0

†Comorbidities: Anxiety and depression; Obesity; Joint Hypermobility; Epilepsy; Migraine; Asthma; Alergic Rhinitis; Renal calculli; Latent Tuberculosis; Tabagism; Substance abuse. ‡Frequency of positive tests over the number of cases tested (16 in ERA group; 9 in SpA group); IQR: inter-quartile range

**Table 2:** Clinical presentation in ERA and Juvenile-onset SpA groups of patients

Onset variables	ERA (*n* = 23)	SpA (*n* = 10)	*p* value
Age at onset (years), median (IQR)	9 (6.5–10.7)	12.5 (10-13.7)	0.01*
Disease duration (years), median (IQR)	3 (2.7–6.2)	16 (12.2–18.7)	0.01*
Time to diagnosis (years), median (IQR)	1.5 (0.5–2.7)	5.5 (2.5-6)	0.01*
N of affected joints at onset, median (IQR)	1 (1–2)	1 (1–1)	0.2
N with upper limbs synovitis, n (%)	0 (0)	0 (0)	1.0
N with lower limbs synovitis, n (%)	21 (91.3)	10 (100)	1.0
Low back pain, n (%)	5 (21.7)	3 [30]	0.7
Enthesitis, n (%)	18 (78.3)	8 (80)	1.0
Symmetric synovitis, n (%)	5 (21.7)	0 (0)	0.2
Systemic signs/symptoms, n (%)	5 (21.7)	4 [40]	0.4

**Table 3:** Disease profile in ERA and Juvenile-onset SpA groups of patients

Variable	ERA (*n* = 23)	SpA (*n* = 10)	*p* value
Disease progression, median (IQR) (months)	9 (2.7–15)	18 [12–33]	0.2
Upper limbs, n (%)	5 (21.7)	5 (50)	0.2
Lower limbs, n (%)	20 (86.9)	10 (100)	0.5
Symmetry, n (%)	8 (34.8)	2 [20]	0.4
Low back pain, n (%)	16 (69.5)	9 (90)	0.4
Inflammatory low back pain, n (%)	11 (47.8)	7 (70)	0.4
SpA shift, n (%)	10 (43.5)	10 (100)	0.01*
Age at Sacroilitis onset, median (IQR) (years)	12 (8.7–14)	16.5 (13.6–18)	0.03*
Time to SpA median (IQR) (years)	2.2 (1-3.7)	4 (1.7–6.7)	0.3
Sacroiliitis on X-ray, n (%)	3 [13]	7 (70)	0.01*
Sacroiliitis on MRI, n (%)	11 (47.8)	5 (50)	1.0

†SpA: Spondyloarthritis; MRI: Magnetic Ressonance Imaging

IQR: Interquartile range

As shown in Table 2, the median age (min-max) at onset for ERA cases was 9 (7-16) years, and the main presentation was lower limbs oligoarticular arthritis or monoarthritis, mostly asymmetric involving the knees (48%) and ankles (30%), 3 with initial arthritis on the feet, and none with hip involvement. Enthesitis at onset was frequent (78.3%), but no spine or sacroiliac joint involvement were observed. Constitutional signs and symptoms, unrelated to Systemic JIA descriptors, were recurrent fever, sore throat, and unspecific maculopapular rash. The median age (min-max) at onset for the juvenile-onset SpA group was 12.5 (19-36) years, with similar joint presentation with lower limb mono/oligoarticular asymmetric arthritis; however, there was a higher frequency of hip (40%) and knee (30%) involvement, and lower frequency of ankles (10%) and none of the feet involvement. The enthesitis frequency was similar, but spine and constitutional features were more frequent at disease onset.

The time gap from symptom onset to diagnosis was significantly higher in the SpA group (5.5 vs. 1.5 years; *p* < 0.03). A low frequency of uveitis and HLA–B27 were identified; however, this biomarker was not systematically tested due to limited availability (31.2% vs. 33.3%; *p* = 0.62). The follow-up and outcome descriptors (Table 3) were: the median time for progression of arthritis was lower in the ERA group than in the SpA group (9 vs. 18 months; *p* = 0.17), although the difference was not significant; 21.7% of the ERA and 50% of the SpA groups, developed upper limb arthritis. Referring to axial involvement, 21.7% of ERA and 30% of juvenile-onset SpA patients presented low back pain at onset, and 69.5% and 90% during disease course, respectively; 43.5% of ERA patients evolved to fit SpA classification, mostly in those with inflammatory pattern history (81,8%).

**Fig. 1:**
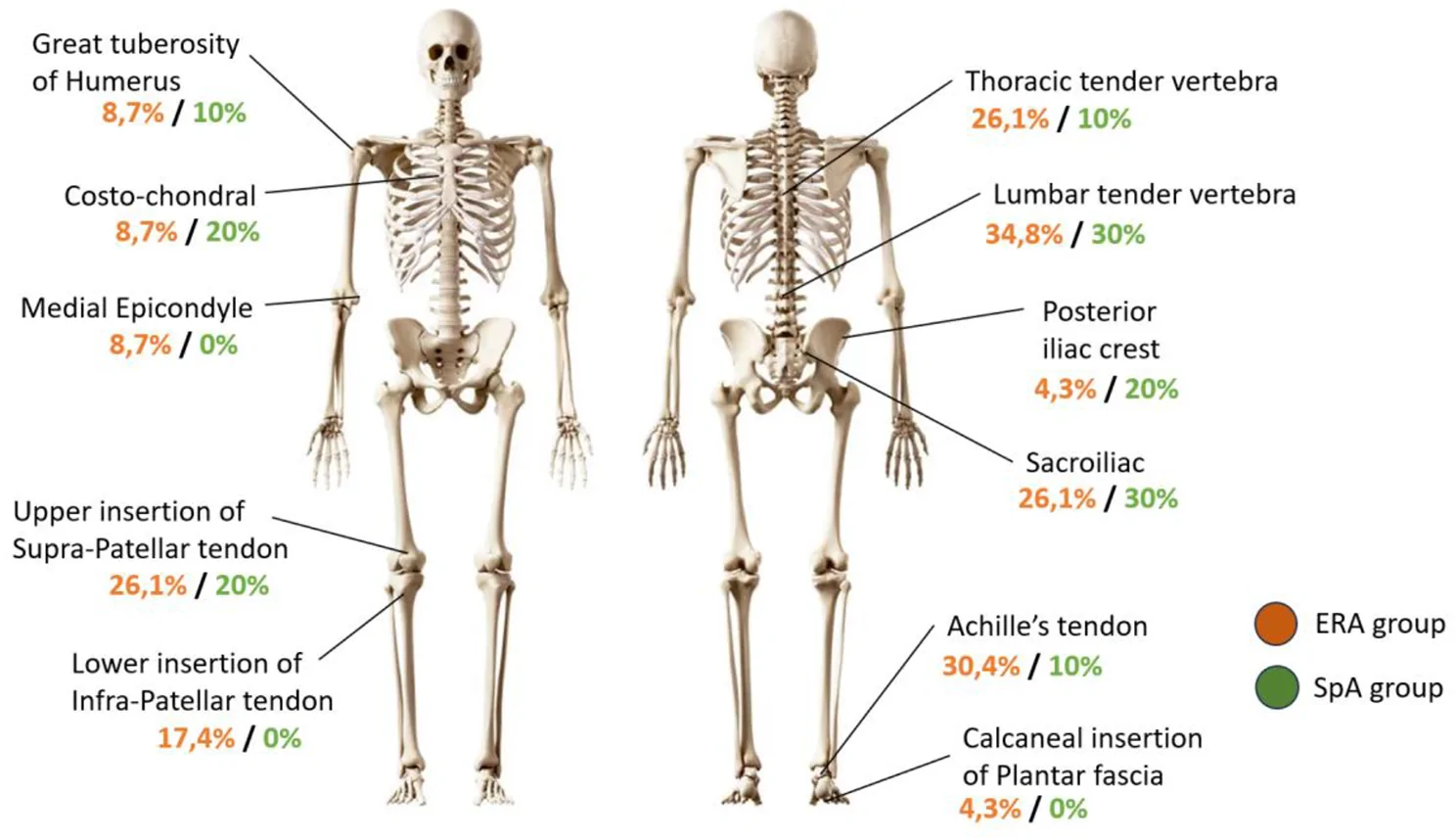
Frequency of the most common painful enthesis sites in ERA vs. Juvenile-onset SpA groups at the cross-sectional evaluation. ^†^ERA: Enthesitis related arthritis; SpA: Spondyloarthritis

The disease activity parameters and respective composite scores for the ERA and SpA groups are presented on Table 4. Overall, they had similar scores, except for nocturnal back pain, where SpA group had higher scores. The enthesitis sites and their frequencies are reported on Fig. 1. They were predominant in the lower limbs, knees, and ankles sites in the ERA group, in contrast to the hips and spine sites in the SpA group.

**Table 4:** Cross sectional assessment of disease activity status in paediatric (ERA) and adult (Juvenile-onset SpA) group of patients

Current status variables	ERA (*n* = 23)	SpA (*n* = 10)	*p* value
N of painful joints, median (IQR)	1 (0–2)	1 (0–2)	0.9
N of swollen joints, median (IQR)	1 (0-1.5)	0 (0–0)	0.2
Frequency of Enthesitis, n (%)	16 (69.5%)	5 (50%)	0.4
Clinical sacroiliitis, n (%)	5 (21.7%)	3 (30%)	0.7
JADAS, median (IQR)	8 (4-13.5)	10.5 (3.1–14.7)	0.7
JsPADA, median (IQR)	2.5 (1.7–3.7)	2.75 (1.7–4.7)	0.6
JADI-A, median (IQR)	1 (0–1)	1.5 (0-3.7)	0.2
JADI-E, median (IQR)	0 (0–0)	0 (0–0)	0.9
VAS spine pain (0–10), median (IQR)	5.5 (1.5-8)	2 (0.2-5)	0.8
VAS night pain (0–10), median (IQR)	1 (0-4.2)	4 (0.2–7.5)	0.07
VAS joint/entheses pain (0–10), median (IQR)	4 (0.7-8)	4.5 (2.2–8.7)	0.4
VAS patient (global) (0–10), median (IQR)	4.5 (1.5–7.5)	6 (2.2–7.5)	0.8
VAS physician (global) (0–10), median (IQR)	2.5 [1–4]	2.5 (0.5-4)	0.8
Schöber test (cm)	14 (13-14.5)	14.4 (14-14.9)	0.5
MASES (0–13), median (IQR)	1.5 (0–3)	1 (0–5)	0.9
LEI (0–6), median (IQR)	0 (0–2)	0 (0–1)	0.4
SPARCC (0–16), median (IQR)	0.5 (0-2.5)	1 (0–1)	0.7

JADAS: Juvenile Spondyloarthritis Disease Activity Score; JsPADA: Juvenile Spondyloarthritis Disease Activity Index; JADI-A: Juvenile Arthritis Damage Index (Articular); JADI-E: Juvenile Arthritis Damage Index (Extra-articular); VAS: Visual analogic scale; MASES: Maastricht Ankylosing Spondylitis Enthesitis Score; LEI: Leeds enthesitis index; SPARCC: Spondyloarthritis Research Consortium of Canada

**Fig. 2:**
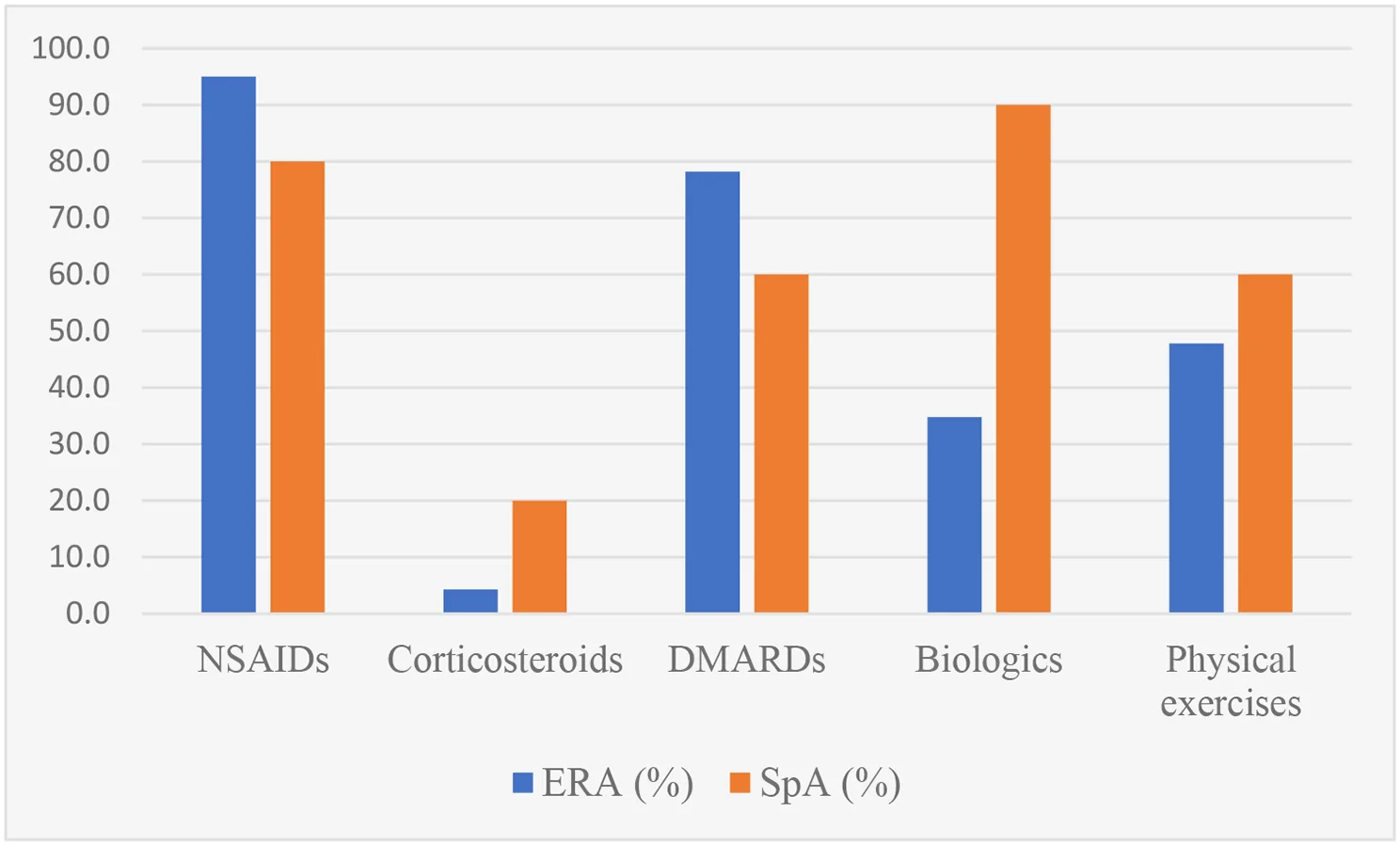
Frequency of cumulative drug treatment over disease course in ERA vs. Juvenile-onset SpA groups of patients ^†^NSAID: Non-steroidal anti-inflammatory drugs; DMARD: Disease-modifying antirheumatic drugs. ERA:Enthesitis related arthritis; SpA Spondyloarthritis

**Fig. 3:**
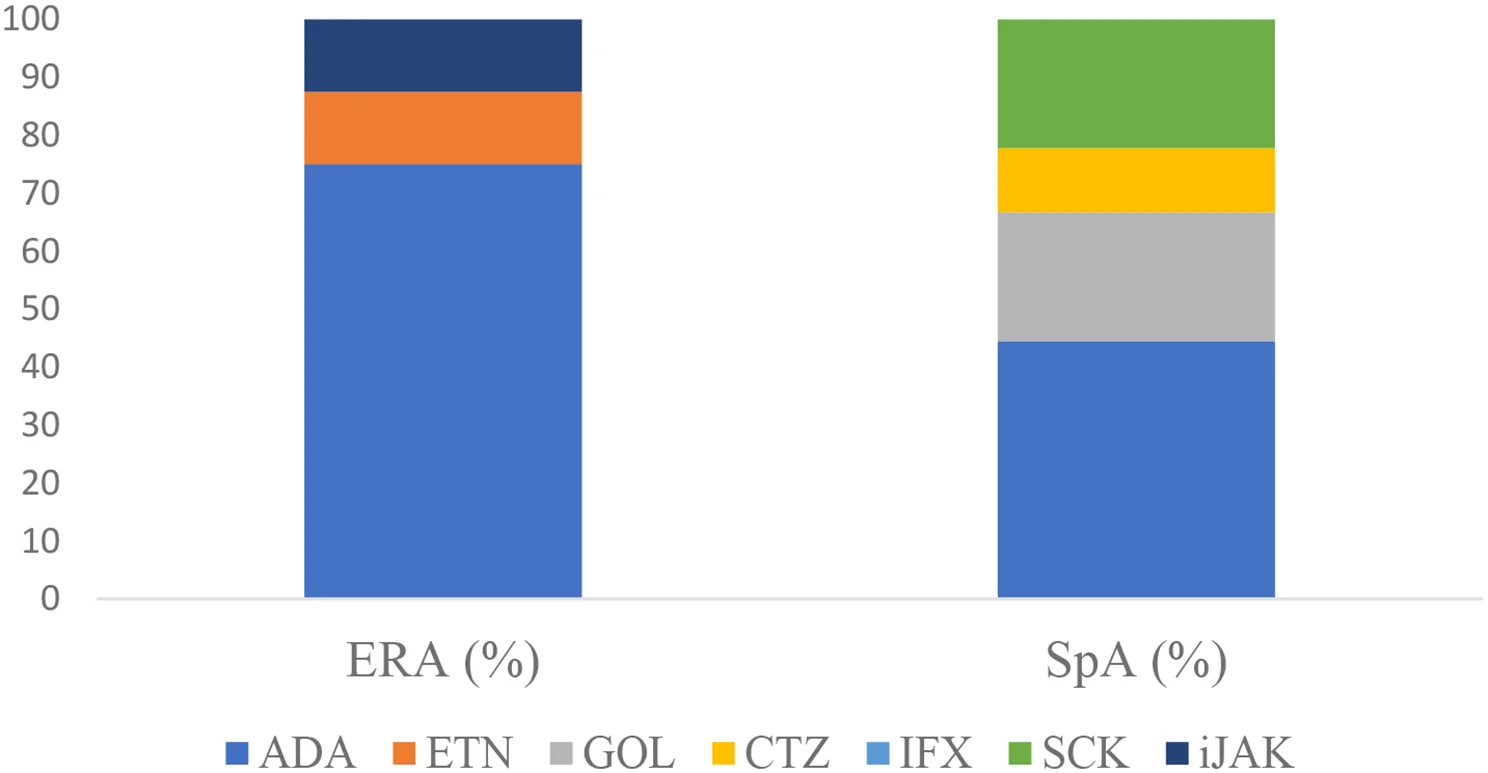
Frequency and type of biologic agents treatment in ERA vs. Juvenile-onset SpA groups of patients, during the disease course. ^†^ADA: Adalimumab. ETN: Etanercept; GOL: Golimumab; CTZ: Certolizumab Pegol; IFX: Infliximab; SCK: Secuquinumabe; JAKi: JAK inhibitor (Baricitinib)

The management approach for ERA and SpA resulted in 91.3% of ERA patients and 100% of SpA patients receiving any medication by the time of cross-sectional assessment. Of those, 39.1% and 30% considered disease activity worsening and 47.8% and 50% with stable disease for ERA and SpA groups, respectively. Cumulative drug treatment frequency throughout disease course is described on Fig. 2. Nonsteroidal anti-inflammatory drugs (NSAIDs) followed by disease-modifying antirheumatic drugs (DMARDs), particularly methotrexate, were more frequently used in the ERA group than in the SpA group, where biologic agents were used more frequently, particularly the anti-TNF alpha agent adalimumab (Fig. 3).

## Discussion

We compared ERA and juvenile-onset SpA patients due to similarities in their clinical features [24]. SpA comprises a wide spectrum of clinical and muscle-skeletal features, evolving to spinal involvement with back pain; this is less frequent in ERA, where arthritis and enthesis are predominant features and spine involvement is more frequently reported in youngsters [25]; such differences could be possibly related to muscle-skeletal development stage, immune response, and the gut microbiota, beyond genetic and other environmental factors.

Our results related to the ERA group of patients and those related to the juvenile-onset SpA group had similar profiles to those reported in previous studies [26–28]: predominance in males, late onset compared to other JIA subtypes, asymmetric lower limbs oligoarthritis, with feet arthritis both in metatarsal joints and toes. In contrast, SpA adults with early onset of symptoms (before 18y) had a higher frequency of hip arthritis, and lower frequency of feet arthritis. Overall, our age-related features results are comparable to previous reports, but to our knowledge there was no comparison in pediatric-adult clinics simultaneously. Our results about uveitis frequency and positive HLA-B27 tests possibly reflect ethnic and geographic differences, as a wide variability of the frequency is reported in the literature [27]. However, we must acknowledge the limitation of the number of performed tests in our routine practice.

The spinal involvement assessments in our series resulted in 69.5% of ERA patients with back pain history, and 47.8% with inflammatory pattern; and radiological axial involvement progression evidence in 43.5%, with being higher in those with inflammatory back pain (81.8%), and a median progression time of 2.3 years and onset age of 12 years. Of note, only 21.7% of ERA patients presented sacroiliitis signs by clinical examination. Previous studies reported up to 34–62% of ERA patients with sacroiliitis by MRI regardless of clinical symptoms, where up to 50% of those develop axial symptoms within 5 years of disease onset, and up to two-thirds develop axial symptoms within 10 years [29–31]. There is no evidence-based recommendation about the right time for imaging screening of sacroiliac/spinal involvement in children and youngsters with ERA, as age-related unmet needs to be addressed. In practice, it is indicated in adolescents, as a strategy to identify patients at risk [32].

Both groups presented a high frequency of enthesitis, especially of lower limbs in the ERA group, and entheses of the pelvic girdle and spine in the juvenile-onset SpA group. The systematic assessment of enthesitis during musculoskeletal exam by the rheumatologist is the most important diagnostic and classification tool and a straightforward direction for guiding treatment, and it is possibly related to practice skills during routine care by the specialist. The standardized enthesitis approach is well established for SpA given the MASES, SPARCC, and MASEI scores [20], but to a lesser extent to pediatrics patients assessments during follow up.

The diagnosis delay was addressed in our series, where ERA had earlier diagnoses than juvenile-onset SpA. This may be explained by more awareness of childhood arthritis, especially the early recognition of ERA and its descriptors for SpA progression in young people. Additionally, population-related sociocultural factors and limited access to care might have influenced the delays on specialist visit for diagnosis and treatment [33]. Previous reports indicated prolonged disease activity and worse outcome in ERA patients compared to other JIA subtypes [34–36], where 45% of the patients scored active disease, needing treatment even after 18 years of follow-up, including those treated with biologic agents. In the present study, the majority of patients were receiving treatment during cross-sectional evaluation. In addition, the recognised risk factors for a worse prognosis in ERA, as the presence of HLA-B27-positive biomarkers, male sex, obesity, ankle arthritis, hips and sacroiliac joints involvement, persistent inflammatory markers, diagnosis delay, incomplete response to treatment and subclinical spinal involvement may also have contributed to disease progression [36].

Concerning treatment, the ERA group treatment profile reflected the current guidelines for JIA treatment [37, 38], with an initial and symptomatic approach with NSAIDs and DMARDs as the main therapeutic strategy guided by peripheral arthritis and enthesitis, and less treatment with biologic agents, indicated only for sacroiliitis and spinal involvement. In the juvenile-onset SpA group, treatment was also aligned with the main guidelines, with a higher proportion and diversity of biologic agents use [39, 40], that might have also been driven by the longer disease course.

We must acknowledge the limitations of a real-world study conducted in the standard of care during the COVID-19 pandemic sanitary restrictions by the time of data collection. The limited access to HLA-B27 and imaging tests, including X-rays and MRI, and the convenience sample might have affected both groups equally. On the other hand, we performed a comprehensive systematic assessment for a representative population coming from 70 towns in large region covered by the national health insurance (SUS) referral.

In conclusion, ERA and juvenile-onset SpA have similar phenotypes with age-related changes and progress over time. Further systematic assessment performed in continuity in pediatric and adult clinics could provide more insights about JIA classification as steps towards proper treatment.

## Data Availability

All data and materials are available from the first author files and the institution’s repository.

## Abbreviations

ADA: Adalimumab
ANA: Anti-nuclear antibodies
ASAS: Assessment in spondyloarthritis international society
CTZ: Certolizumab
DMARDs: Disease modifying anti-rheumatic drugs
ERA: Enthesitis-related arthritis
ETN: Etanercept
GOL: Golimumab
HLA B-27: *Human Leucocyte Antigen* B-27
IFX: Infliximab
iJAK: Janus kinase inhibitor
ILAR: International league of associations for rheumatology
JADAS: Juvenile arthritis disease activity score
JADI-A: Juvenile arthritis damage index - articular
JADI-E: Juvenile arthritis damage index – extra-articular
JIA: Juvenile idiopathic arthritis
JsPADA: Juvenile spondyloarthritis disease activity index
LEI: Leeds enthesitis index
LFN: Leflunomide
MASES: Maastricht ankylosing spondylitis enthesitis escore
MTX: Methotrexate
MRI: Magnetic resonance imaging
NSAIDs: Nonsteroidal anti-inflammatory drugs
OMERACT: Outcome measures in rheumatology
PRINTO: Pediatric rheumatology international trials organization
PYGADA: Physician’s global assessment of disease activity
RA: Rheumatoid arthritis
RF: Rheumatoid factor
SCK: Secukinumab
sJIA: systemic JIA
SPARCC: Spondyloarthritis research consortium of Canada
SpA: Spondyloarthritis
VAS: Visual analog scale
